# Supplementary material for: Using single-cell perturbation screens to decode the regulatory architecture of splicing factor programs
Source: Nucleic Acids Res. 2025 Oct 17;53(19):gkaf855. doi: 10.1093/nar/gkaf855 (PMC12530888; doi:10.1093/nar/gkaf855)
Supplement: gkaf855_Supplemental_Files [file gkaf855_supplemental_files.zip › supplementary_data.docx]

# **SUPPLEMENTARY FIGURES**


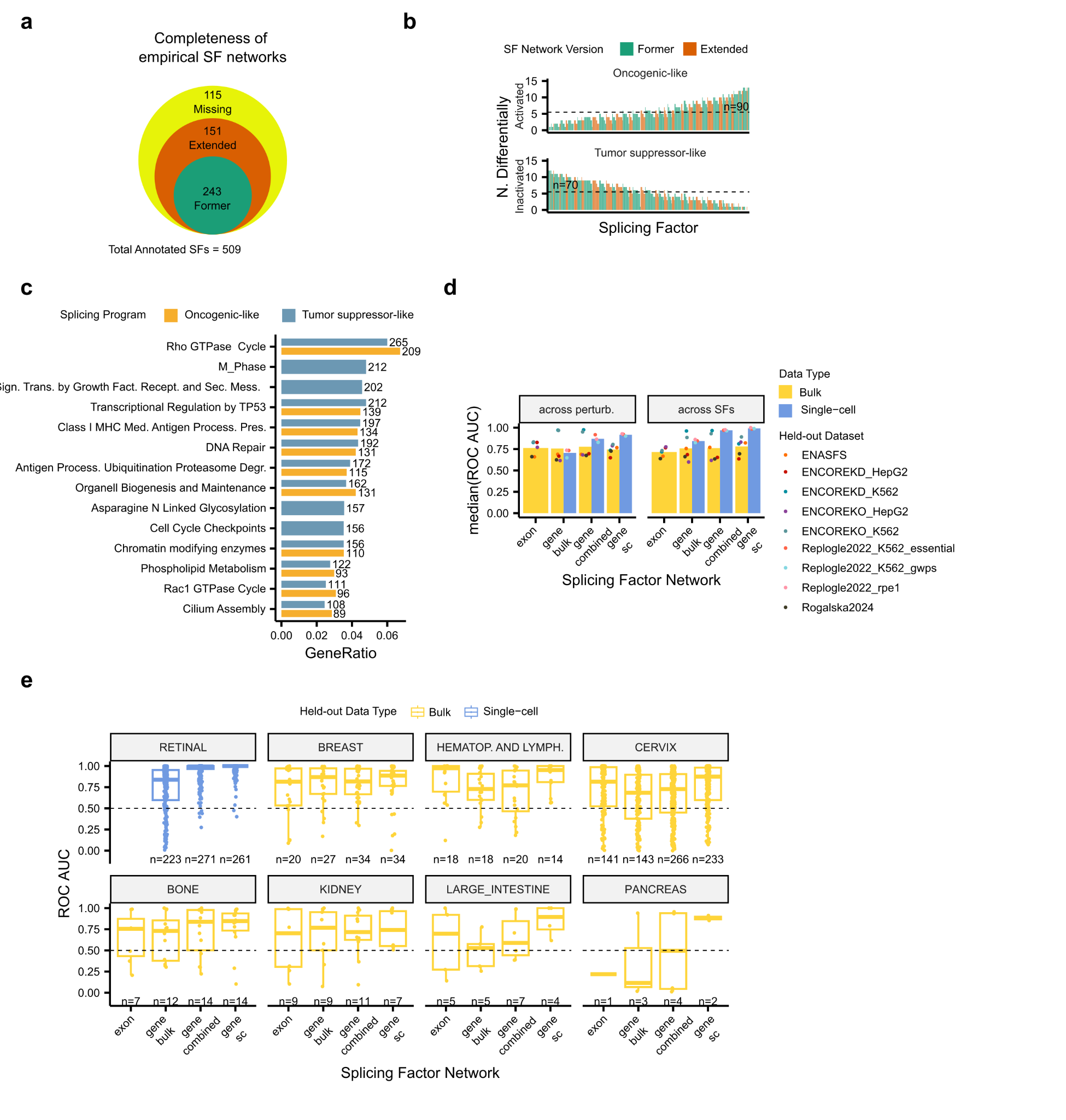


### **Supplementary Figure 1. Benchmarking extended splicing factor networks and redefining cancer splicing programs.**

**(a)** Venn diagram of the number of splicing factors considered in the former and extended versions of empirical splicing factor networks compared to the total of annotated splicing factors.

**(b)** Re-definition of cancer splicing programs with extended splicing factor networks. Number of cancer cohorts in which a splicing factor is recurrently activated (top) or inactivated (bottom). Cancer splicing programs are defined with those splicing factors recurrently active in at least 5 cancer types.

**(c)** Overrepresentation analysis (ORA) of genes corresponding to target exons of splicing factors in re-defined cancer splicing programs. Gene sets from ReactomeDB (n=1,604). We only show the top 10 significantly enriched sets (FDR < 0.05, ORA) sorted by gene ratio combining the enriched gene sets for both splicing programs. A total of 98 and 262 gene sets were enriched for oncogenic-like and tumor suppressor-like splicing factors. Those gene sets missing one cancer splicing program imply that the given gene set was not significantly enriched in the missing program.

**(d)** Leave-one-out evaluations for identifying the experimentally perturbed splicing factor from splicing factor activities estimated using different types of networks and hold-out datasets.

**(e)** Leave-one-out benchmark stratified by cellular lineage using networks from K562 and HepG2 cell lines to estimate splicing factor activity.


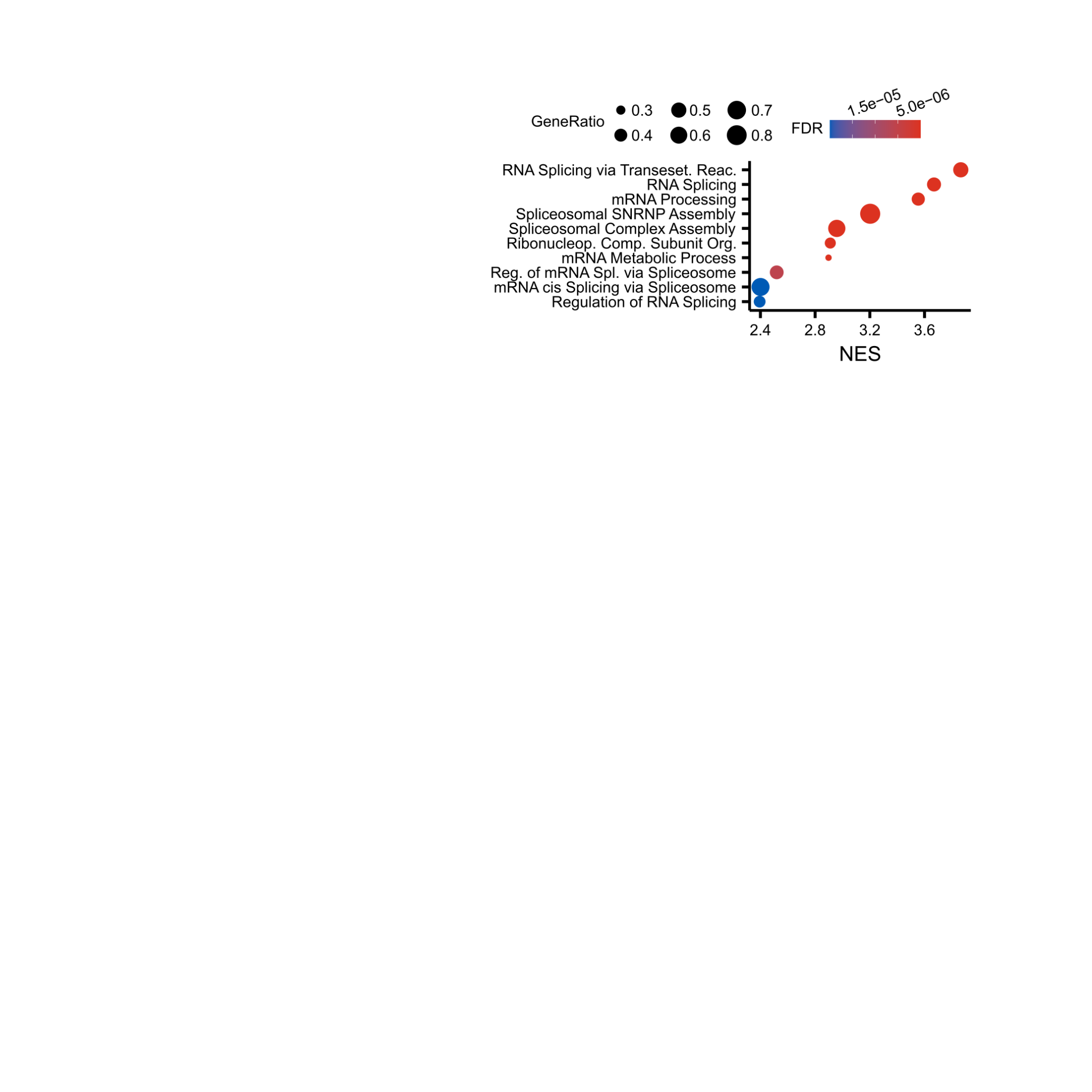


### **Supplementary Figure 2. Enrichments of activity differences between cancer splicing programs.**

Top 10 significantly enriched (FDR < 0.05, GSEA) GO biological processes based on Perturb-seq knocked-down genes sorted by corresponding median activity difference between cancer splicing programs.

### **
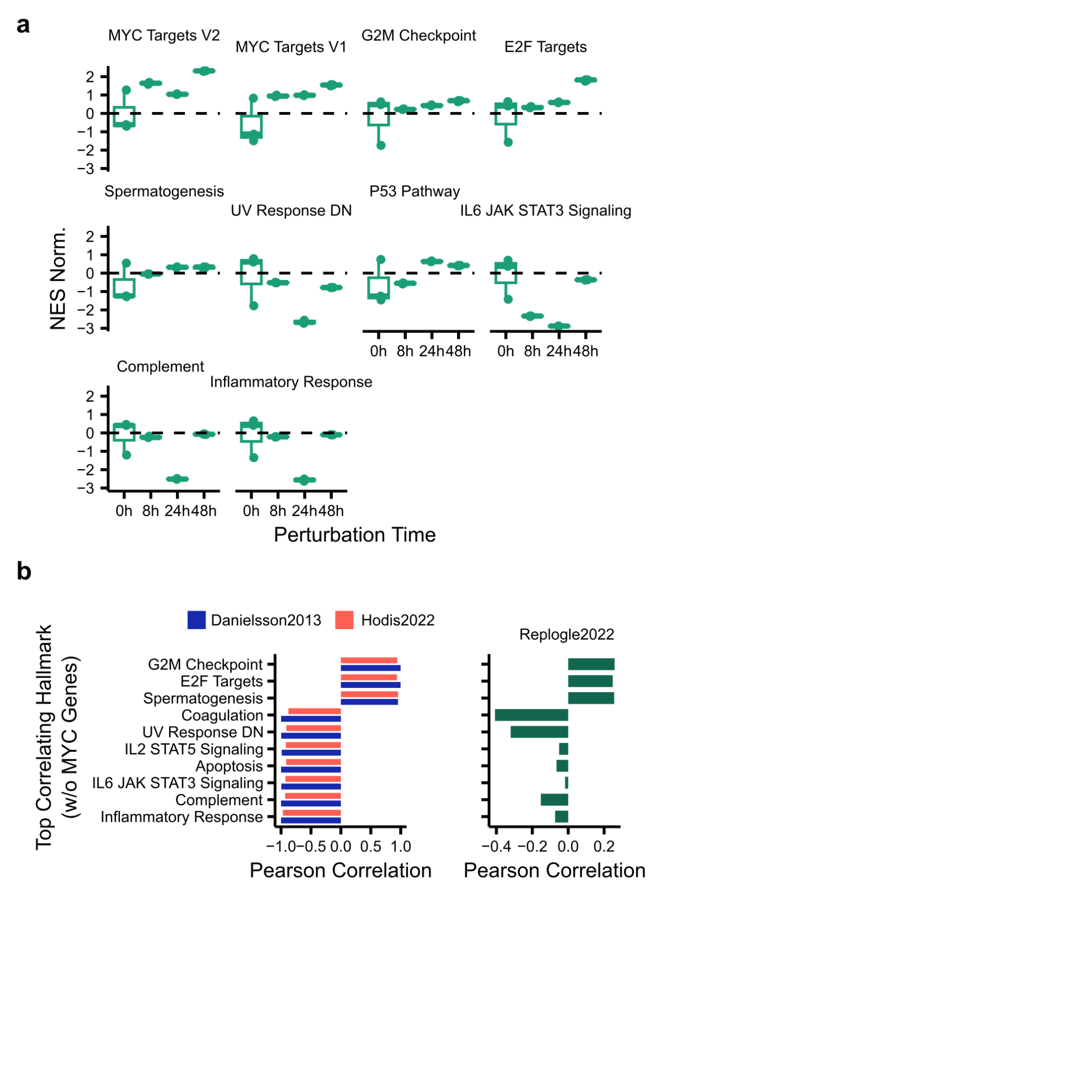
**

### **Supplementary Figure 3. GSEA of changes in expression upon inducing MYC activation and pathway prioritization analysis ignoring MYC target genes.**

**(a)** Normalized NES from GSEAs for each pathway prioritized in Fig. 3b in immortalized MCF10A cells inducibly activating MYC upon 4-OHT treatment. We normalized NES by subtracting NES from the control condition, MCF10A cells without the inducible system treated with 4-OHT for the same time points. In box and whisker plots, the median is marked by a horizontal line, with the first and third quartiles as box edges. Whiskers extend up to 1.5 times the interquartile range, and individual outliers are plotted beyond.

**(b)** Left, top and bottom 5 prioritized pathways performing correlation analysis between program activity differences and NES computed removing MYC genes from the gene ontology across carcinogenesis (Danielsson et al. and Hodis et al.) datasets. Right, correlations across Perturb-seq knockdowns of selected pathways.

### **
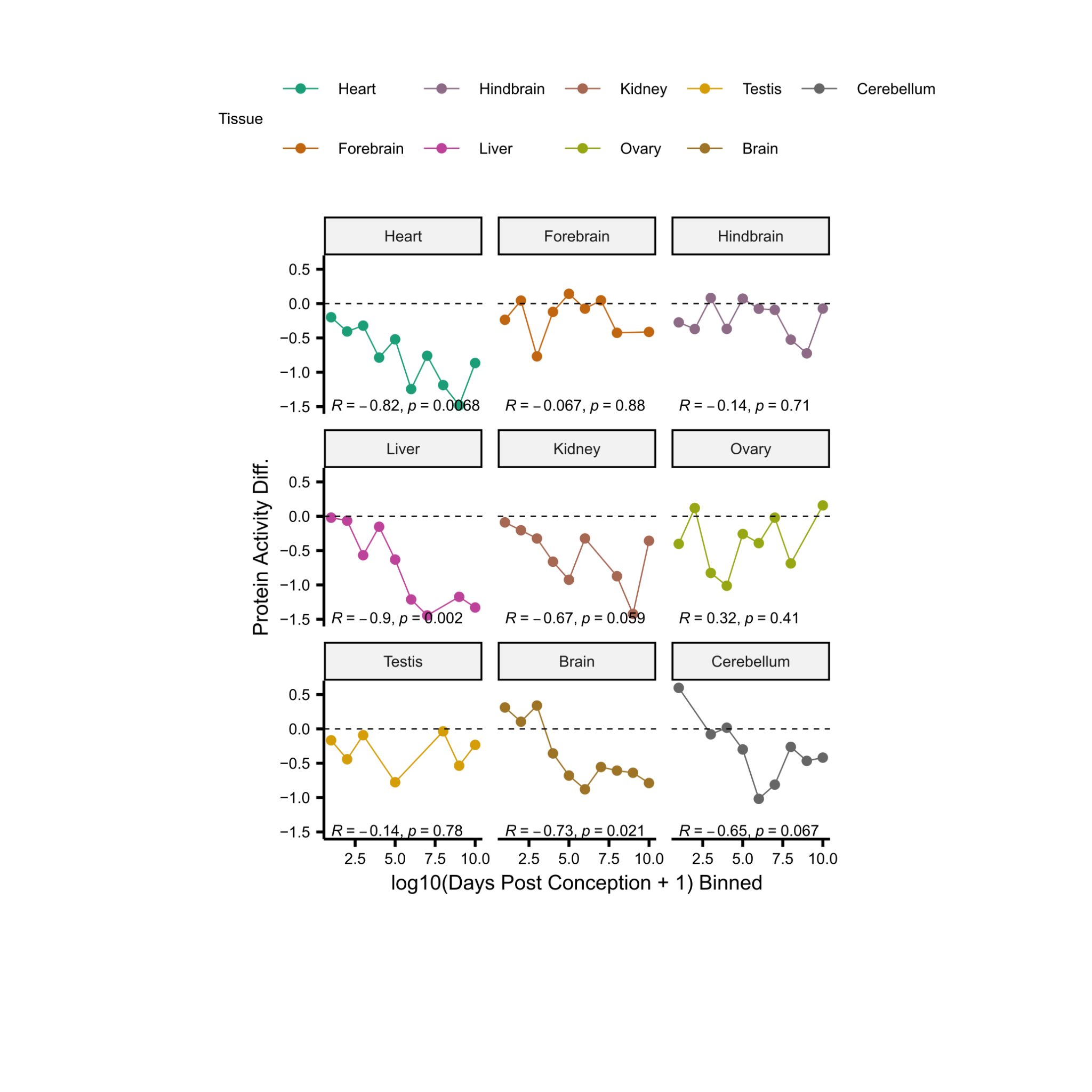
**

### **Supplementary Figure 4. The activity of cancer splicing programs during development split by tissue.**

Labels, Spearman correlation coefficients for each tissue, and corresponding p-values.


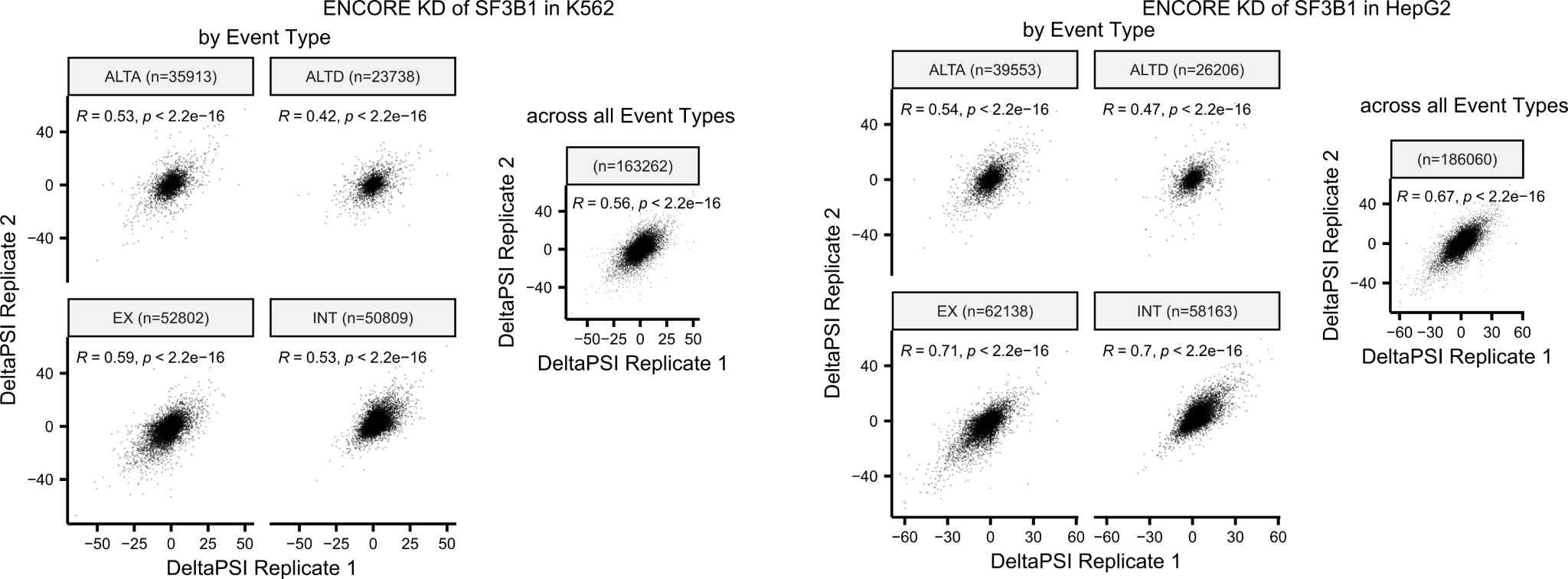


### **Supplementary Figure 5. Among splicing event types, exon inclusion changes upon SF3B1 inactivation yield the most reproducible signatures.**

Relationships between splicing event usage for the two biological replicates perturbing SF3B1 in K562 and HepG2 from the ENCORE project.

# **SUPPLEMENTARY TABLES**

### **Supplementary Table 1. 509 annotated splicing factors.**

### **Supplementary Table 2. Redefined cancer splicing programs.**
